# A National Collaborative Project to Decrease Healthcare-Associated Infections in Intensive Care Units: Results from Stop Hospital Infection, in Portugal

**DOI:** 10.3390/antibiotics15090861

**Published:** 2026-09-03

**Authors:** Rui Malheiro, André Amaral Gomes, Cristina Nunes, Paulo Sousa, Paulo Borem, José-Artur Paiva, Ana Lebre

**Affiliations:** 1 Unidade de Saúde Pública, Unidade Local de Saúde São João, 4200-319 Porto, Portugal; 2 Departamento de Ciências da Saúde Pública e Forenses, e Educação Médica, Faculdade de Medicina da Universidade do Porto, 4200-319 Porto, Portugal; 3 EPIUnit ITR, Instituto de Saúde Pública da Universidade do Porto, 4050-600 Porto, Portugal; 4 Unidade de Cuidados Intensivos, Hospital CUF, 4100-180 Porto, Portugal; 5 Departamento de Medicina, Faculdade de Medicina da Universidade do Porto, 4200-319 Porto, Portugal; 6 Programa de Prevenção e Controlo de Infeção e Resistência aos Antimicrobianos, Direção-Geral da Saúde, 1049-005 Lisboa, Portugal; 7 Grupo Infeção e Sépsis, 4450-681 Matosinhos, Portugal; 8 Serviço de Medicina Intensiva/Serviço de Epidemiologia, Prevenção e Controlo de Infeções e Resistência aos Antimicrobianos, Unidade Local de Saúde do Nordeste, 5301-852 Bragança, Portugal; 9 Centro de Investigação em Saúde Pública, Escola Nacional de Saúde Pública, Universidade Nova de Lisboa, 1600-407 Lisboa, Portugal; 10 Institute for Healthcare Improvement, Cambridge, MA 02138, USA; 11 Serviço de Medicina Intensiva, Unidade Local de Saúde São João, 4200-319 Porto, Portugal; 12 Serviço de Doenças Infeciosas/Unidade Local do Programa de Prevenção e Controlo de Infeção e Resistência aos Antimicrobianos, Instituto Português de Oncologia do Porto Francisco Gentil, 4200-450 Porto, Portugal

**Keywords:** healthcare-associated infections, collaborative, infection prevention and control, intensive care unit, bundle, breakthrough series

## Abstract

Objectives: The collaborative “Stop Hospital Infection 2.0”, implemented in Portugal between 2023 and 2025, aimed to reduce the incidence of catheter-associated urinary tract infection (CAUTI), ventilator-associated pneumonia (VAP) and central-line associated bloodstream infection (CLABSI) by 50.0% in 21 hospitals. Methods: The project followed the Breakthrough Series methodology. Each team was to audit the national preventive bundles, using a Kamishibai card, and drive changes using plan–do–study–act cycles. Before the project, teams reported their 6-month baseline incidence levels. The main outcome was the incidence of infection. The secondary outcome was the proportion of teams that achieved a ≥50.0% reduction in incidence. The number of infections, deaths and bed-days potentially prevented, and costs saved during the project were estimated, along with the potential gains in the future. Results: Incidence rate for teams reporting at least 36 months decreased 47.6% in CAUTI (4.8 to 2.5 infections/1000 device-days), 66.1% in CLABSI (1.5 to 0.5 infections/1000 device-days) and 50.0% in VAP (9.9 to 5.0 infections/1000 intubation-days). The proportion of teams achieving a reduction of at least 50% in infection incidence was 60.0%, 100% and 57.1%, respectively. Teams reporting ≥24 months may have prevented 465 HAI, 108 deaths, 4526 bed-days and saved 8,375,841 euros. In the future, the teams who reported for at least 36 months may avoid 150 cases, 36 deaths, 1538 bed-days and 2,693,546 euros, yearly. Conclusions: A national collaborative focused on evidence-based bundles may be effective in decreasing the incidence of CAUTI, CLABSI and VAP in adult intensive care units.

## 1. Introduction

Healthcare-associated infections (HAIs) are a major problem in patient safety, affecting morbidity, mortality and financial costs to hospitals and health systems [1,2]. Their incidence is significantly higher in Intensive Care Units (ICUs) than in general hospital wards [3]. In the latest report for HAIs in ICUs from 2022, the European Centre for Disease Prevention and Control (ECDC) estimates that 9.8% of patients staying over 48 h in an ICU presented with at least one HAI, considering only those under surveillance—pneumonia, bloodstream infection and urinary tract infection [4].

To challenge this problem, Portugal implemented, from 2015 to 2018, a national collaborative intervention in 12 hospital centres comprising 19 hospitals, named Stop Hospital Infection: A Gulbenkian Challenge, which was able to decrease the incidence rate of ventilator-associated pneumonia (VAP), central-line associated bloodstream infection (CLABSI) and catheter-associated urinary tract infection (CAUTI) beyond the established goal of 50.0% within a three-year period [5]. The project was based on adherence to preventive care bundles, which for each infection have been shown to be associated with better outcomes [6,7,8,9,10,11,12]. However, with the disruption caused by the pandemic, as of 2022, HAI incidence was at levels similar to the 2015 baseline for participating hospitals [4].

In this light, there was a need to implement a new, robust intervention with a similar methodology but a strong focus on sustainability. Following the previous experience, the Portuguese Directorate-General of Health, through its priority program on infection prevention, control and antibiotic resistance (PPCIRA) and with the support of *Fundação Calouste Gulbenkian* and the scientific support of the Institute for Healthcare Improvement (IHI), promoted the second phase of the collaborative “Stop Hospital infection”, which aimed to reduce HAIs by 50% in 21 hospital institutions during a three-year period. We present the implementation process and results of the project in the participating hospitals’ ICUs, using the Breakthrough Series (BTS) model.

## 2. Results

A total of 22 teams reported data on CLABSI and VAP, and 17 on CAUTI. Between 81.8 and 94.1% of the teams reported at least 24 months—6 months of baseline and 18 months of intervention—depending on the typology of infection. Nearly half (between 40.9 and 50.0%) completed the project. The aggregate baseline incidence rate for teams who completed the 36-month duration of the project was 4.8 (confidence interval (CI) 3.5–6.4) infections per 1000 catheter-days in ICU for CAUTI, 1.5 (CI 0.9–2.5) infections per 1000 central line-days for CLABSI, and 9.9 (8.1–12.1) infections per 1000 intubation-days for VAP. These represent the median of the aggregated first six months of reporting. By the end of the project—months 31 to 36 of reporting—these incidence rates had dropped to 2.5 (1.6–3.7) infections per 1000 catheter-days (47.6% reduction), 0.5 (0.2–1.2) infections per 1000 central line-days (66.1% reduction) and 5.0 (3.8–6.6) infections per 1000 intubation-days (50.0% reduction), respectively.

**Figure 1:**
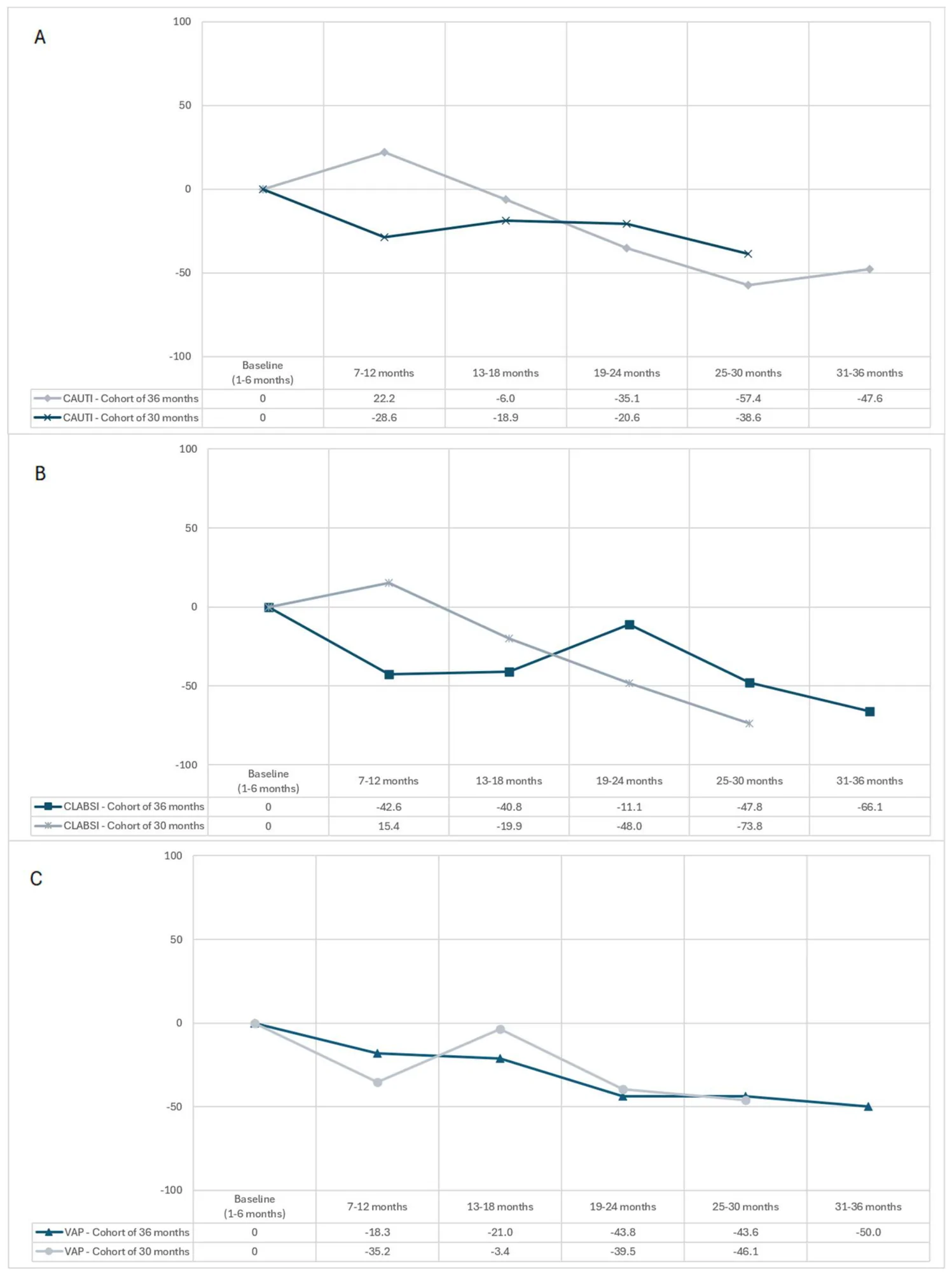
Percentage of reduction in incidence by typology of infection, compared to baseline level, per semester. (**A**) Percentage of incidence rate reduction in catheter-associated urinary tract infection, by cohort. (**B**) Percentage of incidence rate reduction in central line-associated bloodstream infection, by cohort. (**C**) Percentage of incidence rate reduction in ventilator-associated pneumonia, by cohort. CAUTI, catheter-associated urinary tract infection. CLABSI, central line-associated bloodstream infection. VAP, ventilator-associated pneumonia.

Across all infection types, teams showed a long-term decreasing trend in incidence rates. For CAUTI, teams that fully implemented the project initially experienced an increase in incidence, followed by four consecutive semesters of continuous improvement. Although a 50% reduction was achieved by month 30, this result was not sustained thereafter. In the same infection category, teams that initiated participation later demonstrated a more gradual reduction over time. By the end of the project period, the difference in improvement between cohorts was 9.0% (Figure 1A).

For CLABSI, teams in cohorts of 36 and 30 months began with different trajectories. However, in the last 18 months of the collaborative, the trend in decreasing incidence rate was similar between cohorts, and both met the objective of decreasing incidence by 50.0% (Figure 1B).

For VAP, both cohorts showed a decreasing trend across the study period. The reduction was smoother than for the other typologies, with greater variability in incidence rate over time in the 30-month cohort. In the last two months of reporting, across cohorts, the improvement rate was similar, with teams in the 36-month cohort meeting the 50.0% reduction threshold and teams in the 30-month cohort achieving a 46.1% reduction (Figure 1C).

Regarding the secondary outcome, the proportion of teams achieving a reduction of at least 50.0% in infection incidence ranged from 57.1% for VAP to 100% for CLABSI (Table 1). Two teams were excluded from CAUTI analysis, one from VAP and four from CLABSI, due to either insufficient reporting of data points or a baseline incidence of zero combined with the absence of reported days between infections.

During the project, participating teams reporting at least 24 months reported 465 fewer HAIs than expected, of which over half were VAP. From these, estimations indicate that a total of 108 deaths and 4526 bed-days may have been avoided, and 8,375,841 euros saved by the health system, over half of which was due to VAP. In the future, if the teams who completed the project sustain these results, they could be expected to save 150 cases per year, corresponding to 36 deaths avoided, 1538 bed-days freed, and 2,693,546 euros (Table 2).

## 3. Discussion

The collaborative project Stop Hospital Infection 2.0 met its objective of decreasing incidence of CLABSI and VAP by 50% in participating teams completing the project. The incidence of CAUTI also surpassed the 50% threshold in the teams that completed the intervention, but the result was not sustained.

These findings are further supported by the observation that, for CLABSI, teams that initiated the project later achieved a marked reduction in incidence (73.8%), and all teams included in secondary outcome 2 reached the collaborative target. For both CAUTI and VAP, more than half of the participating teams showed reductions of at least 50% in incidence. Although this proportion was higher for CAUTI than for VAP, the collaborative objective at the aggregate level was met only for VAP. This difference may be explained by the greater relative influence of larger hospitals on indicator 1, as improvements in higher-volume institutions contribute more substantially to reductions in aggregate incidence. This consideration supported the inclusion of indicator 2 as a complementary team-level performance measure. The similarity in improvement observed for CAUTI and VAP also illustrates that a 50.0% reduction threshold represents a somewhat arbitrary benchmark, as the magnitude of improvement between these infection types was comparable.

Although the marked improvement in CLABSI incidence may be related to the objectiveness of its criteria [4], experience in other settings cautions against such an interpretation. In Brazil, in a national ICU improvement project with the support of IHI, which also followed the BTS, the most marked incidence reduction was for CAUTI (65.8%), followed by VAP (52.1%) and CLABSI (43.5%) [13]. In a quasi-experimental study in five ICUs from Recife, a subgroup analysis of the same project showed a non-significant before-and-after comparison in CLABSI incidence rate, whereas moderate improvement was achieved in the other typologies [14]. Interestingly, while at the national level there seemed to be a negative association between bundle adherence and incidence rate, that was not the case in Recife, where the improvement appeared to be associated with the decreased use of indwelling devices [13,14].

Other studies with the same principle—improving bundle adherence to decrease HAI incidence—yielded variable results, ranging from 74% to 96% improvement in CLABSI and 15.0% to 43.6% in VAP [15,16,17]. The difference may be partially attributed to differences in project implementation, adherence of professionals, different baseline and potential for improvement, variable processes for selecting participants, leadership, and broader organizational factors that may impact healthcare results. However, one should not neglect the role of heterogeneity in the bundles adopted. In a systematic review of the impact of bundle application on CLABSI incidence rate, although the results suggest a significant positive association, the heterogeneity in adult ICUs is 91.0% [9]. Although bundles are designed based on the best available evidence [18], each study selects a different number and combination of items. The same heterogeneity is found in published care bundles for the prevention of VAP [10,19].

The convergence in outcomes between the cohorts participating for 36 months and those participating for 30 months from a given semester onward suggests a possible spillover effect of the intervention. Although implementation occurred at different time points and progressed at different rates across sites, teams that began reporting later may have been indirectly influenced by the ongoing implementation in other hospitals. This effect would not be explained by bundle adherence, but rather by an increased awareness of the HAI problem, participation in meetings with other teams, and an overall improved culture of safety, which may be difficult to measure. Although this effect has been described for community interventions such as vaccination and mass drug administration [20], it is conceptually possible that it may be applicable in the context of non-simultaneous health interventions.

To the best of our knowledge, although other studies have shown relevant savings in economic costs at local settings [21,22], this is the first study to estimate the potential number of infections prevented, lives saved, and the impact on healthcare delivery in terms of length of stay and financial costs within a national collaborative project. A key finding is that, even under a worst-case scenario assuming no sustainability of the Stop Hospital Infection 2.0 intervention, at least 108 lives were potentially saved during the study period. Although the estimated cost savings do not account for downstream resource use following bed availability—i.e., subsequent admissions and associated costs—it should also be noted that broader economic benefits, including productivity gains, improvements in healthcare delivery, and reductions in morbidity, are likewise not captured in the financial estimates.

This study has several limitations. Teams failed to provide a simultaneous baseline and effectively began the intervention at different times. This hinders our ability to assess the impact of the intervention without contamination, which is why we chose to divide the analysis into cohorts, with the cohort of teams reporting at least 36 months free from that effect. It is possible that teams in this cohort may have significantly different aspects from teams in the other cohorts. The variability in the measures of baseline, with only six points available, limited our ability to perform a before-and-after study using an interrupted time-series, which would have produced more robust results. Likewise, issues such as regression to the mean or seasonal effects were not possible to be assessed. It is also possible that changes may have been due to concurring interventions, although the sustainability and evolution of the incidence-rate time trend suggest otherwise.

As mentioned earlier, another limitation is that larger hospitals have a larger weight on indicator 1. The delineation of a 50% aggregate improvement in the incidence rate at the national level could lead to greater focus on tertiary hospitals. The same differential weight applies to the point estimation of lives, bed-days and financial costs saved. The absence of at least one tertiary hospital from that analysis means that some millions were not added to our final estimate. We also emphasise that bed-days and financial costs are dependent on the published literature. Some estimates may not closely reflect the actual costs in our setting. Likewise, the estimated impact of a similar intervention varies across health systems, thereby limiting the external validation of our findings. Finally, data on PDSA performed and adherence to process indicators were insufficiently reported in *SimpleQI* to allow meaningful analysis. Therefore, we were unable to show whether our results were associated with an improvement in bundle adherence, per typology.

## 4. Methods

### 4.1. Study Design and Intervention

This is a quality improvement report, assessing the implementation of the collaborative project “Stop Hospital Infection 2.0” in Portuguese ICUs, from January 2023 to December 2025. The collaborative followed the BTS methodology, described in detail elsewhere [18]. Succinctly, it comprises several core components: topic selection informed by real-world data, recruitment of expert faculty, enrolment of participating organisations and teams, structured learning sessions involving both teams and experts, interspersed action periods, and a model for enhancing and implementing improvement activities. The purpose of the collaborative was to promote a culture of safety in the National Health System, through the implementation of a series of evidence-based practices aimed at preventing HAIs.

The project was implemented in two phases. In the planning phase, the governance structure of the programme was defined, comprising a faculty and an executive committee, mentors and subject-matter experts. A driver diagram and a structured change package were developed for each care bundle, which were published as national guidelines [23,24,25]. Local leaders received comprehensive training in leadership, improvement science, measurement methodologies and Training Within Industry (TWI). In the quality control phase, systematic and continuous monitoring of infections was implemented. Each team was to audit each item of the preventive bundle using a Kamishibai card (K card), which has been shown to facilitate and sustain bundle adherence in infection control [26]. The auditor would mark the K card green if the item was well applied, or red if it was missed or inappropriately applied. All cards were placed on a Kamishibai board, standing on a wall and visible to everyone, so that it would be immediately apparent which items were being well prosecuted and which ones required improvement. To support the standardisation of processes, each item had a process instruction sheet detailing what was needed to comply with the item, which were complemented by targeted TWI sessions. For the items requiring improvement, teams were coached to drive changes using plan–do–study–act (PDSA) cycles during the action periods. Furthermore, strategic huddles and site visits were performed, checklists and structured rounding guides introduced as key patient safety tools, and Pareto charts implemented to perform structured problem analysis.

The proposed goal of Stop Hospital Infection 2.0 in ICU teams was a 50.0% reduction in the baseline incidence of CAUTI, CLABSI and VAP. The incidence of each HAI was defined as the number of new cases of the specific HAI in the numerator, divided by the number of device-days in the denominator, following the European Centre for Disease Prevention and Control definitions [4]. For each HAI, a driver diagram was developed to support local interventions, stating the overall goal, the interventions of the bundle, and additional relevant change ideas that were to be implemented or promoted.

Before implementing changes, teams were instructed to report their incidence levels for the last six months. Data were reported monthly, and the six months were used to calculate a median baseline incidence value, which served as a reference throughout the project. When the median baseline incidence was zero, teams were instructed to use a different outcome indicator, namely the sum of device-days exposure between two consecutive infections. With this indicator, the goal was to obtain at least 1000 device-days between any two infections [27].

Data on adherence to bundle components were collected and submitted via the online surveillance platform *SimpleQI^®^.* Data on disease incidence was reported in the same platform, drawing run charts that were used to redefine the median, according to statistical process control methodologies. Participating teams had permission to input and modify their own data and could also access benchmarking data from other participating hospitals. The platform additionally provided standardised definitions of process and outcome indicators, together with detailed guidance regarding inclusion and exclusion criteria. Although teams were allowed to develop and report locally defined indicators within the platform, these indicators were not included as part of the national collaborative dataset and were therefore excluded from this analysis.

### 4.2. Participants

The Portuguese Directorate-General of Health, through the program on infection prevention, control and antimicrobial resistance (PPCIRA/DGS), promoted the project through an open call, made at the national level, for all public hospitals with adult ICUs to participate. The application was voluntary, and no financial incentive was provided.

The inclusion criteria were public hospitals with at least 200 adult beds and an ICU, and the leadership of the hospital’s administration board had to provide formal written willingness to participate. In line with these criteria, 22 teams from 21 hospitals participated in the project on ICU-related HAI, reporting VAP and CLABSI. Of these, only 17 teams reported on CAUTI. A hospital team refers to a group of nurses and doctors (usually three) working in a specific ICU service, ward, or group of beds spanning more than one ward. Hence, one hospital could have more than one team participating, and the same team could start reporting each HAI at different points in time.

### 4.3. Outcomes

The major outcome of this study was the aggregated incidence of infection according to the typology of infection.

The secondary outcome was the proportion of teams, by infection typology, that achieved at least a 50.0% reduction in incidence density. Furthermore, the number of infections and deaths prevented during the project period, together with the corresponding cost savings, were estimated. Projections were also made regarding the expected annual number of infections, deaths and costs that could be avoided in the future if the observed results are sustained at the national level.

### 4.4. Statistical Analysis

Although the project was initially designed to include a six-month period of simultaneous participation to establish a pre-intervention baseline incidence density of VAP, CLABSI, and CAUTI across participating ICUs, with the intervention scheduled to begin in June 2022, implementation varied across settings in real-world conditions. Participating teams effectively initiated the project at different times. Therefore, for each ICU, the six-month baseline period was defined locally by the teams and corresponded to different calendar periods. For aggregation purposes, baseline data were aligned by relative time, such that month 1 of the baseline period from one ICU was combined with month 1 from another ICU regardless of the corresponding calendar date. Accordingly, the intervention phase was considered to begin at aggregated month 7. Only six time points were included in the aggregated baseline period. When teams reported a longer baseline period, only the last six months of baseline were considered. Data on cases (numerator) and device-days (denominator) were added, per reporting month, and an aggregated national incidence density calculated, per month. A significance level of 0.05 was considered for confidence intervals.

**Figure 2:**
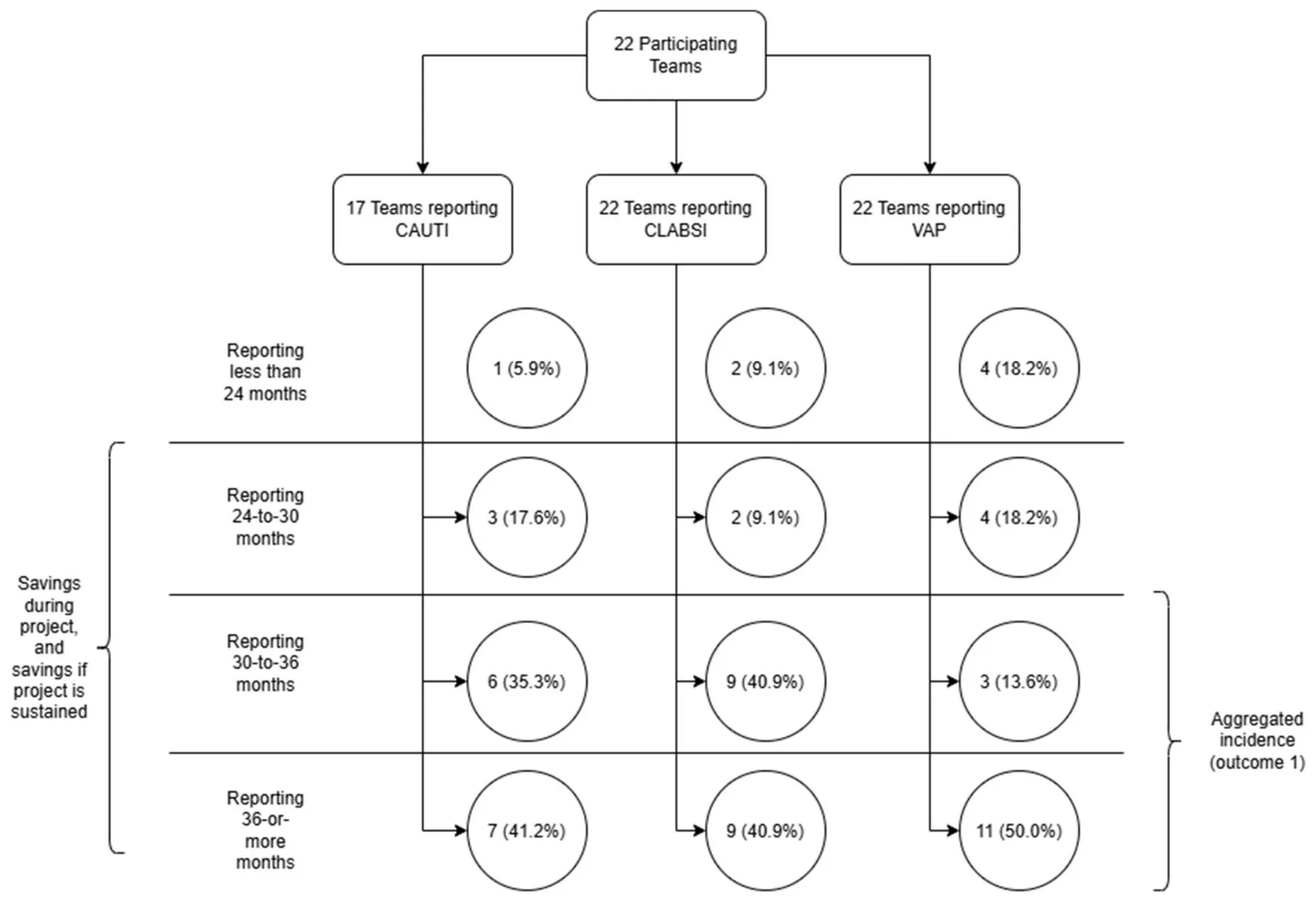
Flow chart of participating teams, cohorts and teams, with specification of outcomes 1 and 3. CAUTI, catheter-associated urinary tract infection. CLABSI, central-line associated bloodstream infection. VAP, ventilator-associated pneumonia.

Since teams began the project at different dates, not all of them reached the 36 months of reporting initially intended. Therefore, teams were divided into three cohorts based on the number of reporting months: one for teams reporting the entire 36 months, one for teams reporting at least 30 months but not 36, and one for teams reporting 24 months but not 30. This was done per typology of infection (Figure 2).

For the analysis of the primary outcome, only teams contributing data for at least 36 months of a specific HAI were included, as these institutions were considered to have fully completed their participation in the collaborative. The baseline level was defined as the median of the aggregated observations from the first six months of reporting for each team. Achievement of a ≥50.0% reduction was evaluated by comparing this baseline level with the median incidence density observed during months 30–36, representing the last six-month interval with complete reporting across all teams in the cohort. The aggregated incidence was also estimated for teams reporting between 30 to 36 months, to compare the evolution of cohorts throughout time, per typology of infection.

For the secondary outcome, all teams were included if they reported at least 12 months (6 from baseline and 6 after the intervention began). For each team, the selected baseline was the median of the 6 months referred to as such by the team in *SimpleQI^®^*, after validation by the national team. Improvement was assessed by comparing the baseline with the latest median on *SimpleQI^®^*, following best practices for run charts in statistical process control [28]. Teams with zero baseline incidence were included if they reported the number of device-days between infections at any point in time. Teams with zero median incidence and no report on days between infections were excluded from this analysis. The analysis was separated by typology of infection.

All cohorts were included in the estimation of infections and deaths prevented, along with the cost savings achieved during the project period (Figure 2). The number of infections prevented was estimated as the difference between the observed infections and the number expected under a contrafactual scenario in which the baseline incidence remained unchanged. Therefore, expected infections were calculated by multiplying the number of infections reported during the baseline period by the number of semesters reported per cohort. These calculations were performed separately for each cohort and infection type. The same contrafactual scenario was considered for the estimation of potential deaths avoided, bed-days freed and financial savings. The number of deaths prevented was estimated by multiplying the number of infections prevented by infection-specific case-fatality ratios reported in the literature [29,30,31]. Financial savings were estimated by multiplying the number of infections prevented by the expected cost per infection type, based on published estimates [32,33,34]. Extra hospital bed-days were estimated by multiplying the number of prevented infections by the expected extra length of stay, in days [33,34,35]. Because costs vary according to health system characteristics and population context, preference was given to studies conducted in Portugal or referring to costs in the Portuguese setting. When such studies were unavailable, estimates from peer-reviewed European studies were used; if these were also unavailable, cost estimates from studies conducted in other settings were considered. The values on which the analysis was based are presented in Table 3.

To estimate the potential savings assuming sustained performance following completion of the collaborative, the number of infections reported during the final six months of participation was subtracted from the number reported during the baseline period. This difference was interpreted as the number of infections expected to be prevented in each subsequent six-month interval if the reductions achieved during the collaborative are maintained relative to the pre-intervention situation. The corresponding numbers of deaths prevented and the cost savings were estimated by multiplying the projected number of infections prevented by infection-specific case-fatality ratios and cost estimates, as described above. The analysis was stratified by typology of infection.

## 5. Conclusions

A national collaborative focused on evidence-based care bundles may help drive large-scale reductions in the incidence of healthcare-associated infections in adult ICUs across three infection types. Even suboptimal adherence to the collaborative may be associated with substantial estimated financial savings and reduced mortality. Future initiatives may draw on these findings to design robust interventions at the national level.

## Figures and Tables

**Table 1:** Proportion of teams with at least 50% reduction in the incidence of infection, by typology of infection.

Typology of Infection	Participating Teams	Included Teams	Teams with Improvement of at Least 50%	Proportion of Teams Improving at Least 50%
CAUTI	17	15	9	60.0
CLABSI	22	18	18	100
VAP	22	21	12	57.1

CAUTI, catheter-associated urinary tract infection. CLABSI, central line-associated bloodstream infection. VAP, ventilator-associated pneumonia.

**Table 2:** Estimates of infections prevented, deaths avoided, hospital bed-days saved and costs saved during the collaborative project Stop Hospital Infection 2.0, and the yearly savings in the future, if teams who completed the project sustain their results.

Timing	Outcomes	CAUTI	CLABSI	VAP	Total
Savings during the project	Prevented Cases	127	102	236	465
	Deaths avoided	10	28	71	108
	Hospital days saved	508	714	3304	4526
	Costs saved	377,317	3,050,718	4,947,806	8,375,841
Yearly savings if project is sustained	Prevented Cases	38	26	86	150
	Deaths avoided	3	7	26	36
	Hospital days saved	152	182	1204	1538
	Costs saved	112,898	777,634	1,804,014	2,693,546

Savings during and after project include cohorts of 24, 30, and 36 months. CAUTI, catheter-associated urinary tract infection. CLABSI, central line-associated bloodstream infection. VAP, ventilator-associated pneumonia.

**Table 3:** Case-fatality, costs (in euros) and length of stay (in days) of healthcare-associated infections, per typology.

HAI	Case-Fatality	Additional Cost (Euros)	Additional Length of Stay (Days)
CLABSI	0.269	29,909	7
VAP	0.3	20,965.28	14
CAUTI	0.0789	2971	4

CAUTI, catheter-associated urinary tract infection. CLABSI, central line-associated bloodstream infection. HAI, healthcare associated infection. VAP, ventilator-associated pneumonia.

## Data Availability

Data used for this research belong to Direção-Geral de Saúde. Therefore, they may be available upon reasonable request. Conditions and restrains governing data access are an exclusive responsibility of Direção-Geral da Saúde.
